# PLUS: Predicting cancer metastasis potential based on positive and unlabeled learning

**DOI:** 10.1371/journal.pcbi.1009956

**Published:** 2022-03-29

**Authors:** Junyi Zhou, Xiaoyu Lu, Wennan Chang, Changlin Wan, Xiongbin Lu, Chi Zhang, Sha Cao

**Affiliations:** 1 Amgen Inc., Thousand Oaks, California, United States of America; 2 Department of Biostatistics and Health Data Science, Department of Medical and Molecular Genetics and Center for Computational Biology and Bioinformatics, Indiana University School of Medicine, Indianapolis, Indiana, United States of America; 3 Department of BioHealth, Indiana University–Purdue University Indianapolis, Indianapolis, Indiana, United States of America; 4 Department of Electrical and Computer Engineering, Purdue University, Indianapolis, Indiana, United States of America; University of Michigan, UNITED STATES

## Abstract

Metastatic cancer accounts for over 90% of all cancer deaths, and evaluations of metastasis potential are vital for minimizing the metastasis-associated mortality and achieving optimal clinical decision-making. Computational assessment of metastasis potential based on large-scale transcriptomic cancer data is challenging because metastasis events are not always clinically detectable. The under-diagnosis of metastasis events results in biased classification labels, and classification tools using biased labels may lead to inaccurate estimations of metastasis potential. This issue is further complicated by the unknown metastasis prevalence at the population level, the small number of confirmed metastasis cases, and the high dimensionality of the candidate molecular features. Our proposed algorithm, called **P**ositive and unlabeled **L**earning from **U**nbalanced cases and **S**parse structures (**PLUS**), is the first to use a positive and unlabeled learning framework to account for the under-detection of metastasis events in building a classifier. PLUS is specifically tailored for studying metastasis that deals with the unbalanced instance allocation as well as unknown metastasis prevalence, which are not considered by other methods. PLUS achieves superior performance on synthetic datasets compared with other state-of-the-art methods. Application of PLUS to The Cancer Genome Atlas Pan-Cancer gene expression data generated metastasis potential predictions that show good agreement with the clinical follow-up data, in addition to predictive genes that have been validated by independent single-cell RNA-sequencing datasets.

## Author summary

Metastasis is the major cause of cancer-related deaths, and evaluations of metastasis risk are essential for tailored treatment of cancer patients. Existing methods often build a classifier using the clinical metastasis diagnoses as binary responses or detect genomic features significantly associated with metastasis-related survival outcomes. However, these methods tend to identify genomic predictors that have little consistency across different cancer types. Thus, there is an urgent need for a powerful tool to characterize the cancer metastasis potential applicable across a wide span of cancer types. Computational assessment of metastasis potential based on large-scale transcriptomic cancer data is challenging because metastasis events are not always clinically detectable, which results in biased estimations of metastasis potential. Our proposed algorithm, called PLUS, considers patients with metastasis diagnosis as positive instances and the remainder as unlabeled instances, meaning they are either metastatic or non-metastatic. Such a classifier given by PLUS rendered concordance between the predicted cancer metastasis and observed metastasis survival outcomes in the follow-up data for almost all cancer types considered. The selected genes were found to perform functions consistent with experimental research findings and are capable of clustering the single cells based on their levels of metastasis potential.

This is a *PLOS Computational Biology* Methods paper.

## Introduction

Metastatic cancer is responsible for over 90% of all cancer deaths [1,2]. Compared with well-confined primary tumors, metastatic cancer remains incurable because of its systemic nature and the resistance of disseminated tumor cells to existing therapeutic agents [3,4]. Hence, for a substantial number of cancer patients, effective treatment is largely dependent on an understanding of and capacity to interdict metastasis. Cancer metastasis is a multistep process by which cancer cells disperse from a primary site and progressively colonize distant organs. This process is often schematized as a sequence of discrete steps, termed the invasion-metastasis cascade [5–7]. Although advances have accelerated dramatically over the past decade and provided valuable insights regarding the molecular changes in the process of metastasis, metastatic cancer still represents an emerging field replete with major unanswered questions. In this context, evaluating a cancer patient’s metastasis potential is vital for clinical decision-making and understanding the biological mechanism of metastasis is the first step towards targeted therapeutics.

Previous work has provided strong evidence indicating that a number of genomic markers in primary tumors are associated with the propensity of a patient to develop metastatic relapse, and that distant metastasis events can be inferred from gene expression profiles within the primary tumor bulk [8,9]. For example, a recent study used machine-learning techniques to determine metastatic tumour organ of origin using the somatic mutation data [10]. Several studies have defined gene expression signatures that predict overall and metastasis-free survival as well as progression and metastatic growth in breast cancer patients [11–15]. Kikuchi et al. identified 40 metastasis-related genes by comparing lymph node-positive and lymph node-negative lung cancer patients [16]. Schell et al. [17] developed a score that can separate metastatic and non-metastatic tumors. Klein et al. [18] used the Cox multivariable proportional hazard model and survival C-index to evaluate the ability of a genomic classifier to predict metastasis and validated its robust performance. Goossens-Beumer et al. [19] performed differential MicroRNA (miRNA) expression analysis between metastatic and non-metastatic cases to establish miRNA-based metastasis risk predictions. Md Jahid et al. [20] proposed a personalized approach for improving the prediction of breast cancer metastasis.

On one hand, many of these metastasis predictors have been developed for a certain cancer type and thus are less generalizable to other cancer types. In fact, the molecular signatures of cancer metastasis reported in different studies hardly overlap [21]. Recently, the development of high-throughput sequencing technology has produced a large amount of molecular data at the pan-cancer level. Hence, harnessing the power of large-scale projects such as The Cancer Genome Atlas (TCGA) would enable us to systematically study the abnormalities in cancer progression at the molecular level in a statistically more powerful manner. On the other hand, to develop a cancer metastasis predictor, the following challenges remain unsolved by existing methods, which may largely limit the ability to predict early metastasis events and derive biological insights: 1) Metastasis events are not easily detectable, especially when we consider the hibernating disseminating cancer cells; thus, clinical metastasis diagnoses often tend to underestimate metastatic events. For classification-based methods, training a classifier with under-detected metastatic instances may lead to an under-estimated metastasis potential. 2) Many survival-based studies were principally designed to detect molecular markers that can best predict patient overall, progression-free, or metastasis-free survival, instead of directly targeting metastasis events itself. Therefore, the detected markers may not have any functional implication in metastasis. 3) High-dimensional molecular features complicate the classification and feature selection process. Therefore, a substantial refinement of the statistical considerations for model training and marker identification is required in order to increase the power to predict metastatic potential at the pan-cancer level. In summary, the combination of under-detected metastasis events and high dimensionality in molecular features in transcriptomics data presents both statistical and computational challenges.

We have developed an algorithm called **P**ositive and unlabeled **L**earning from **U**nbalanced cases and **S**parse structures (PLUS) to address the aforementioned challenges. The ultimate goal of PLUS is to enable early metastasis event prediction at the pan-cancer level, as well as to infer biologically meaningful gene markers for metastasis potential. PLUS belongs to a category of classifiers called positive and unlabeled learning (PU learning). Whereas the input to a binary classifier normally consists of positive and negative incidence sets, in PU learning, a learner has access to only positive and unlabeled incidences, and it is assumed that the unlabeled data can contain both positive and negative incidences [22]. PLUS is particularly well-suited for studying metastasis potential: only patient samples diagnosed as metastatic are available and trustable, called positive samples; the samples that are not diagnosed as metastatic, due to a short follow-up time, are either metastatic or non-metastatic and are thus categorized as the unlabeled samples. In addition, PLUS is built on a penalized likelihood estimation framework for variable selection, and its iterative bootstrapping procedure makes it robust to bias caused by unbalanced allocations of positive and unlabeled samples.

PLUS represents a first-of-its-kind method to specifically address the under-diagnosis issue in studying cancer metastasis potential using the PU learning framework. Its robustness enables the power of big data to be harnessed through integration of large-scale datasets collected from different cancer types. Insights gleaned from this research will prove useful to the early diagnosis and treatment of metastatic disease. We benchmarked PLUS on extensively simulated data sets and demonstrated the superiority of PLUS over all other PU learning methods across all simulation scenarios. Application of PLUS to TCGA pan-cancer gene expression dataset resulted in metastasis potential estimations consistent with the clinical follow-up data. Moreover, PLUS selected a set of genes that are highly predicative of metastasis potential, and the differentiating potency of these genes was validated on independent single-cell RNA-sequencing (scRNA-seq) datasets, as well as existing literature.

## Results

### Problem formulation and methods overview

**Fig 1:**
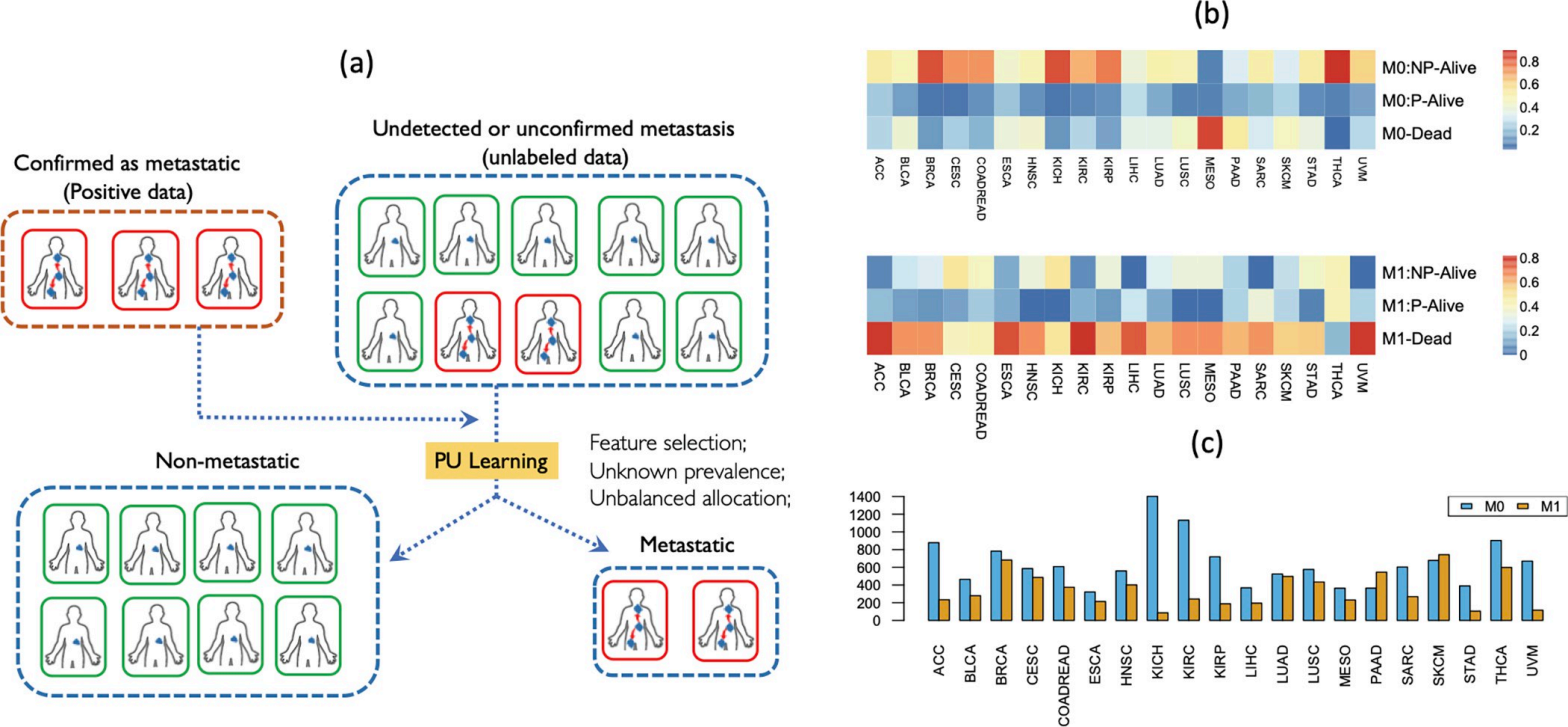
The motivation of PLUS. (a) Under-diagnosis occurred among patients who were not diagnosed as metastatic. To predict the metastasis potential for non-metastatic samples, PLUS builds upon the PU learning framework and is specifically designed to recognize the bias in under-diagnosis, and to address the computational challenges in feature selection, unknown prevalence, and unbalanced allocation. (b) For patients who were clinically diagnosed as non-metastatic (M0) at baseline for each cancer type (columns) in TCGA Pan-Cancer study, the top three rows show the proportions of patients with follow-up information who were found alive and with non-progressed disease (NP-Alive), alive and with progressed disease (P-Alive), and deceased (Deceased). The bottom three rows show the same proportions for patients who were diagnosed as metastatic (M1) at baseline. (c) The median follow-up time (*y*-axis) for patients who were diagnosed as non-metastatic (blue) and metastatic (yellow) at baseline for each cancer type (*x*-axis).

Diagnoses of metastatic cancer are often confirmed by detection of tumor masses at a distant site or effusions on clinical examination or by imaging [23]. Unfortunately, there is currently no panel of basic tests that can aid in revealing metastatic tumor events. Hence, many patients that are not diagnosed with metastatic tumors may have developed metastasis, but could not be diagnosed at an early phase due to weak symptoms (Fig 1A). Take the cancer patients enrolled in the TCGA project as an example. Among patients initially diagnosed as non-metastatic (M0), a large portion have a good prognosis and do not develop metastasis (M0: NP-Alive in Fig 1B). However, a significant portion of these patients do develop metastasis (M0: P-Alive in Fig 1B) or die (M0: Deceased in Fig 1B) based on their follow-up data. This is especially true for such cancer types as BLCA, ESCA, HNSC, LIHC, LUAD, LUSC, MESO, PAAD, SKCM, and STAD. This trend indicates a possible under-detection of metastasis at initial diagnosis. Given the relatively short follow-up time of these cancer types (Fig 1C), we believe that this discrepancy may be even greater if we considered longer follow-up data. In contrast, among patients who are initially diagnosed as metastatic, a majority develop metastasis (M1: P-Alive in Fig 1B) or die (M1: Deceased in Fig 1B) based on the follow-up diagnosis. This trend indicates that metastasis diagnoses, but not the non-metastasis diagnosis, are often trustable. These observations are the motivation of our proposed PLUS algorithm. PLUS is built upon the framework of PU learning, where patients diagnosed as metastatic and non-metastatic are treated as positive and unlabeled instances, respectively. Abbreviations for cancer types, and their initial diagnosis frequencies are given in S1 Table.

PLUS builds upon the case-control framework proposed in [24], and the modeling formulation of PLUS is detailed in the METHODS AND MATERIALS section. Different from many other PU-learning algorithms, the solution of PLUS is achieved by incorporating both an EM-type algorithm and a bootstrap technique tailored for three main challenges that are particularly important for predicting cancer metastasis: (1) The genes that are informative in differentiating the true metastatic and non-metastatic classes are a sparse set of the whole transcriptome, representing a *sparse structure*. (2) The observed positive incidences (M1 diagnoses) are largely outnumbered by the unlabeled samples (M0 diagnoses), indicating *observation unbalancedness*. (3) One class (true metastatic patients) may be much larger or smaller than the other class (true non-metastatic patients), indicating *population unbalancedness*. Unfortunately, the true metastasis prevalence is usually unavailable. Specifically, for (1), we introduce a LASSO penalty into the objective function to select informative features [25]. For (2), we recursively bootstrap from the unlabeled instances of equal size to the positive instances to maintain a relatively high information ratio for the subsequent analyses. For (3), a sigmoid transformation is applied to the probability function in the EM procedure to account for the population unbalancedness.

The general idea of PLUS is that we first bootstrap a subset of unlabeled samples equal in size to the positive set and perform a one-step EM-type procedure to generate the estimated probability for the unlabeled samples. We then repeated this procedure with another sample. The whole process stops when the assigned probability for unlabeled samples is stabilized in the recursive process. Ultimately, the algorithm provides the predicted probability of a sample being positive, denoted as the metastasis potential, as well as genes that may be predictive of metastasis potential.

### Method validation on synthetic data

Using extensive simulated datasets, we compared PLUS with four other state-of-the-art PU-learning algorithms, namely, PUlasso [26], Ada-KNN, Ada-Logit, and Ada-SVM [27], as well as three popular binary classification methods, including the penalized logistic regression (PLR), XGBoost, and random forest. The input of each method includes the covariate matrix *X* (simulated gene expression matrix) and an observed response or label for either positive instances (purely true positive, corresponding to an M1 diagnosis) or unlabeled instances (consisting of true positive and true negative, corresponding to an M0 diagnosis). The output is the predicted probability of a sample being positive (i.e., metastasis potential) for each sample. See the METHODS AND MATERIALS section for details of each method.

Basically, the covariate matrix *X* and true response *y* are linked by a logit model. In addition, to mimic the sparseness of gene features, the covariate matrix is simulated to contain both truly predictive covariates, as well as a large number of noisy features that do not contribute to the prediction of the response. To mimic the under-detection of metastasis, a certain portion of positive instances are randomly selected to be flipped to negative, resulting in an observed negative set that consists of both true negatives and true positives. Both PLUS, PUlasso, and PLR are based on a logit model, and as a wrapper, AdaSample is capable of implementing logistic regression as its core. The XGBoost and random forest approaches are powerful tree-based methods for handling non-linear relationships between the high-dimensional predictors and responses. Hence, simulation data based on the logit model would have a minimal bias towards any methods. The simulation procedure is detailed in the METHODS AND MATERIALS section. We evaluated the prediction accuracies of the methods in various simulation environments, where four parameters are altered: (i) the ratio of true positive and negative instances in the population, which is usually unobservable in real data scenarios, (ii) the level of separation of the two classes, or the noise level, where a higher noise level means that classes are less separable; (iii) the level of unbalancedness for observed positive and unbalanced instances, and (iv) the number of informative covariates among all features.

We assessed the prediction accuracy of all methods on only the unlabeled instances, which contain both true positive and negative instances. The average Area Under the ROC curve (AUC) calculated using the true labels was obtained from 100 repetitions of each simulation setting as the metric for methods evaluation. A higher AUC indicates a better prediction. Here, PLR was applied to provide an “oracle” estimation, as we intentionally provided the PLR with the true positive and negative label information, while the remaining methods were all provided with positive and unlabeled information. Hence, the performance of the PLR served as an oracle prediction that can be made if the given labels are all correct.

**Fig 2:**
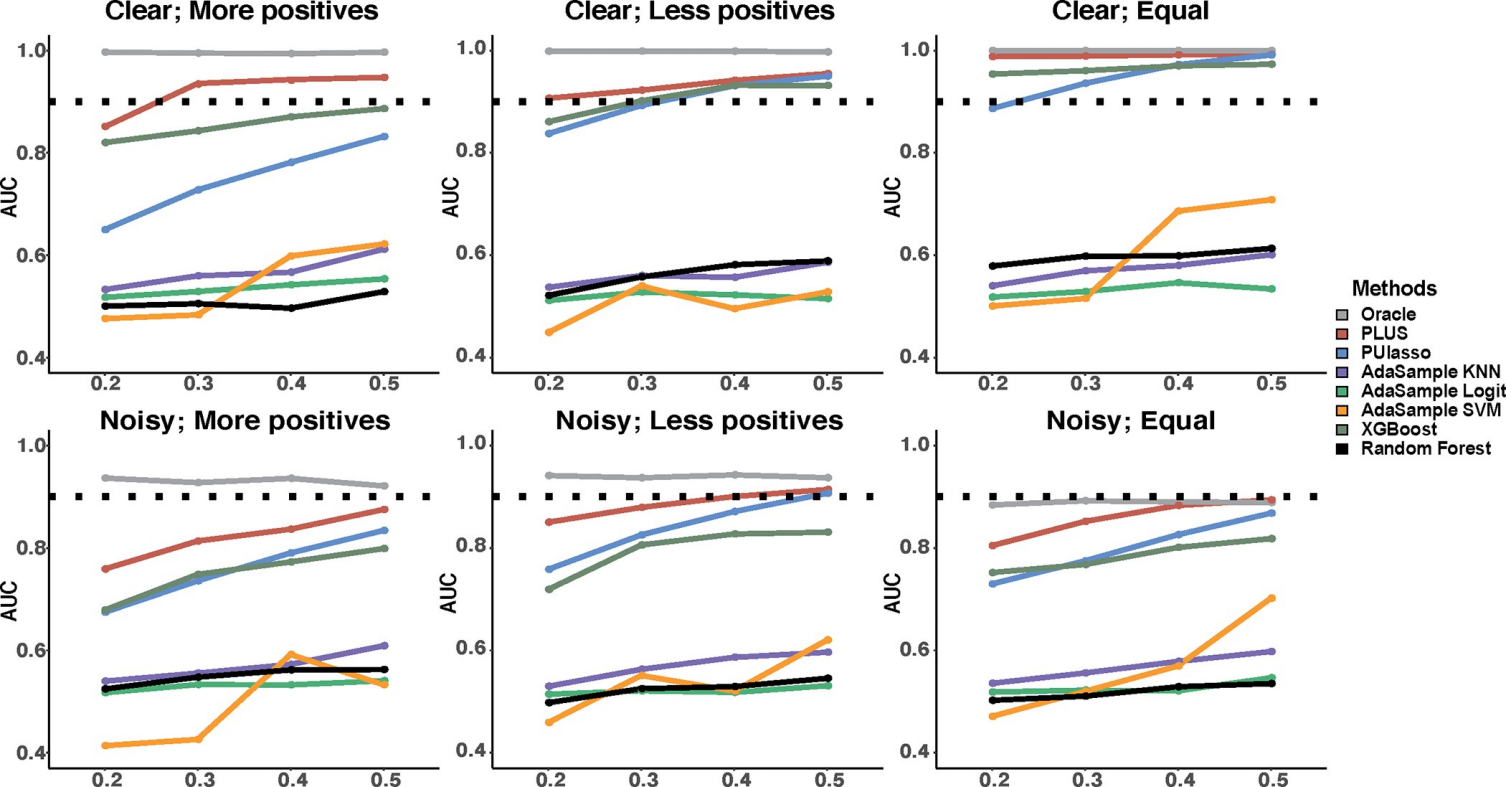
Performance comparisons of the competing methods on simulated data. The top panel shows results for simulation settings in which the positive and negative sets are clearly separable, while the bottom panel shows results for simulation settings in which the two sets are less separable. The left-most column shows results for simulation settings in which there are more true positive samples than true negative samples, the middle column shows results for less true positive samples than true negative samples, and the right-most column shows results for an equal number of true positive and negative samples. The dotted line corresponds to an AUC of 0.9. Here, the *y*-axis represents the AUC. And *x*-axis shows levels of observation unbalancedness, where a larger number indicates that the number of observed positive samples is closer to that of the unlabeled samples, with 0.5 indicating that the numbers are equal.

Fig 2 compares the performance of the PLUS, PUlasso, Ada-KNN, Ada-Logit and Ada-SVM, XGBoost, and random forest, as well as the reference method, PLR, which was provided with the true labels, under different settings: (1) Different levels of observation unbalancedness. On the *x*-axis of each figure, a higher ratio indicates that the number of observed positive samples is closer to that of the unlabeled samples, with 0.5 indicating that the numbers are equal. (2) Different levels of the population unbalancedness. From the left-most to middle to right-most images, we show results for simulation settings with more true positive than true negative samples, more true negative than true positive samples, and equal true positive and true negative samples. (3) Different noise levels of the logit model. From top to bottom, we show results for simulation settings in which the true positive and true negative sets are well-separated (top) and not well-separated (bottom).

In summary, the three Ada- methods and the random forest all performed substantially worse than the other methods, with an average AUC of less than 0.6 for all simulation parameter settings. This result is probably due to the inability to handle high-dimensional features for Ada- methods or to handle unlabeled cases for the random forest, worsened by the population and observation unbalancedness issues. In general, all methods tend to have a higher AUC as the ratio of the observed positive samples increases (change on the *x*-axis in Fig 2). Among the methods, PLUS is the most robust to the ratio of the true positive samples, as it is specifically designed to handle the population unbalancedness issue with a bootstrapping procedure. In contrast, the performances of PUlasso and XGBoost fall sharply as the ratio of observed positive samples decreases. When the true positive and true negative sets become less separatable, all the methods tend to perform worse; here, PLUS still maintains an AUC above 0.8 for almost all scenarios, while PUlasso and XGBoost drop well below 0.8 for most cases. Interestingly, all tested methods tend to achieve the best performance when the ratio of true positive samples is lower (middle column), while they have the worst performance for a higher ratio of true positive samples (left-most column). This trend is reasonable because a higher rate of true positive samples corresponds to a higher contamination of true positive samples in the unlabeled cases, making it more difficult to achieve an accurate estimate on the distribution of true negative samples among unlabeled cases. After all, the best scenario for a prediction arises when all unlabeled cases are truly negative, with the least contamination of true positive samples. When we varied the ratio of informative features (see S1 Text), our analysis suggested that the performances did not vary much for PLUS or PUlasso; however, this parameter moderately affects the performance of XGBoost and the random forest, and severely impacts the performance of the AdaSample methods. This result arises from the built-in model selection capability for PLUS and PUlasso whereas AdaSample methods cannot handle high-dimensional features, and the random forest and XGBoost are known to suffer from scalability/memory issues with high-dimensional features.

Overall, our analysis clearly suggests that PLUS achieves the best performance under all parameter settings over the other tested methods. The AUC of PLUS, averaging over 0.8 for all settings, is closest to the optimal AUC obtained by PLR. Notably, in our real data analysis on TCGA dataset shown in the next section, we have much fewer observed positive samples and a high-dimensional set of features, even though we do not know the ratio of true positive samples. Hence, we expect that PLUS will perform better than the other methods.

### TCGA pan-cancer data analysis

Next, we applied PLUS to the transcriptomic profiles of all 7,467 cancer samples from 20 cancer types in the TCGA cohort. Among these, only 12 cancer types have at least 10 samples confirmed as metastatic at initial diagnosis, totaling 553 samples across the 12 cancer types. These 553 samples are treated as our observed positive samples, while the remainder are treated as unlabeled samples. Details on data pre-processing and sample metastasis diagnosis are provided in the METHODS AND MATERIALS section and S1 Table. We applied PLUS, PLR, PUlasso, and XGBoost to this pan-cancer dataset. All four methods obtained the estimated metastasis potential as the probability of being metastatic for all the samples. Note that because we cannot observe whether metastasis developed in each patient, validating the classification accuracy using the ROC curve is impossible. Instead, we evaluate the performance of the methods on this real dataset by examining the association between the predicted metastasis potential with the progression-free survival (PFS) extracted from the TCGA clinical follow-up data, using only those patients that were initially diagnosed as non-metastatic at the time of tumor tissue collection. The event of disease progression is defined in the METHODS AND MATERIALS section. The predicted metastasis potentials obtained by the four methods for all samples, as well as the PFS data for each sample, are provided in S2 Table.

**Fig 3:**
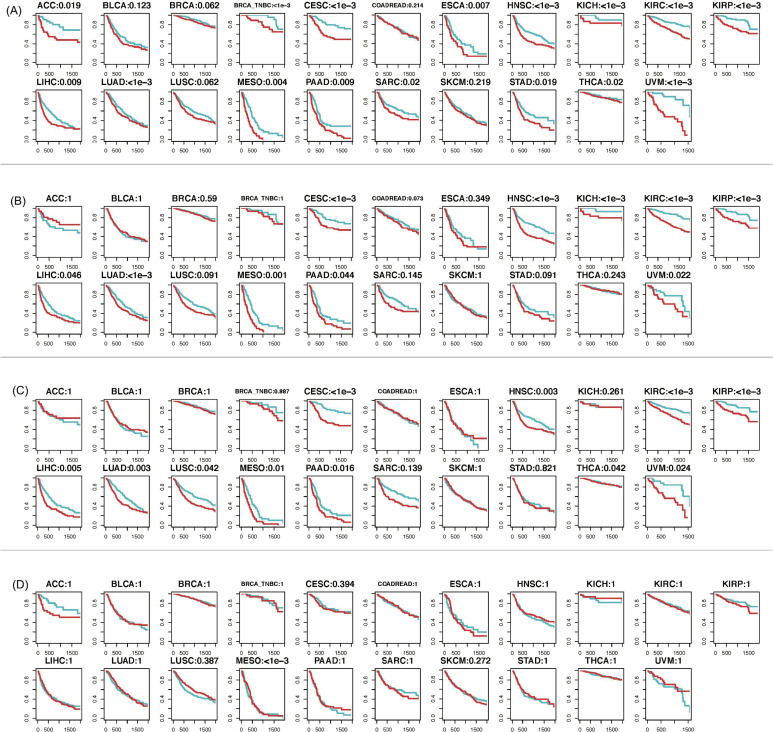
Progression-free survival curves of patients with different metastasis potentials. Higher metastasis potential (red) and lower metastasis potential (blue) for 21 cancer (sub)types are predicted by (A) PLUS; (B) PLR; (C) PUlasso; and (D) XGBoost. The *x*-axis represents time in days and *y*-axis represents the percentage of patients without metastasis event at a particular time point. Note, the *p*-value shown above each figure panel is calculated between the predicted ***continuous*** metastasis potential with the progression-free survival (PFS); the blue and red curves shown in the figure, however, represent patient groups that are ***dichotomized*** at the median of the predicted metastasis potential.

Recognizing that different cancer types have different baseline metastasis potentials, we conducted an association analysis of between patients’ PFS data and their predicted metastasis potential given by each method. The association analysis was conducted using the Cox proportional-hazards model considering continuous predictors [28]. The significance of associations between PFS and predicted metastasis potential, given by PLUS, PUlasso, PLR and XGBoost, is presented in Table 1, and the *p*-values were adjusted for multiple comparisons via the Holm method [29]. Clearly, 16 cancer types showed a significant association between the PLUS-predicted metastasis potential with PFS (p-value < 0.05), with higher predicted metastasis potentials related to worse survival outcomes. For BRCA and LUSC, we observed marginally significant associations (p-value < 0.08). Notably, for BRCA, we observed a significant association for its most aggressive subtype, namely, the triple negative breast cancer (TNBC) (p-value < 0.001). We did not observe significant associations between PLUS prediction and PFS for BLCA, COADREAD, or SKCM. We argue that the follow-up time for BLCA and COADREAD is too short (see Fig 1C), with the follow-up occurring before a metastasis event could be confirmed. Overall, we have demonstrated that for patients initially diagnosed as non-metastatic, PLUS is able to predict the metastasis potential for these patients and detect possible under-diagnosis incidences; moreover, its predicted metastasis events are in strong accordance with true metastasis events based on the follow-up data. For PLR, PUlasso, and XGBoost, strong associations were identified between the predicted metastasis potential and PFS in 9,10, and 1 cancer types, respectively. To visually compare the performances of the four methods, we also compared PFS for high and low metastasis potential groups within each cancer type. Specifically, for each cancer type, we divided the patients (initially diagnosed as non-metastatic) into two equal-sized groups, one with high metastasis potential and another with lower potential, according to each method. Fig 3 shows a survival comparison between the two groups for all 20 cancer types and 1 subtype based on PLUS and the three other methods. Our results and comparisons clearly demonstrate the advantage of applying PLUS for predicting metastasis potential, owing to its robustness in the PU learning setting, with possible unbalancedness in sample collection and population incidence.

**Table 1:** Significance of the association between PFS and metastasis potential predicted by PLUS (column 3), PLR (column 4), PUlasso (column 5) and XGBoost (column 6) using only the patient samples not diagnosed as metastatic (M0 samples). The *p*-values are adjusted for multiple comparisons. The second column shows the number of M0 samples for each cancer type.

	M0 sample	PLUS	PLR	PUlasso	XGBoost
ACC	62	**0.019**	1.000	1.000	1.000
BLCA	346	0.123	1.000	1.000	1.000
BRCA	1054	0.062	0.590	1.000	1.000
BRCA_TNBC	111	**<0.001**	1.000	0.887	1.000
CESC	294	**<0.001**	**<0.001**	**<0.001**	0.394
COADREAD	528	0.214	0.073	1.000	1.000
ESCA	167	**0.007**	0.349	1.000	1.000
HNSC	514	**<0.001**	**<0.001**	**0.003**	1.000
KICH	64	**<0.001**	**<0.001**	0.261	1.000
KIRC	452	**<0.001**	**<0.001**	**<0.001**	1.000
KIRP	278	**<0.001**	**<0.001**	**<0.001**	1.000
LIHC	367	**0.009**	**0.046**	**0.005**	1.000
LUAD	490	**<0.001**	**<0.001**	**0.003**	1.000
LUSC	495	0.062	0.091	**0.042**	0.387
MESO	84	**0.004**	**0.001**	**0.010**	<0.001
PAAD	174	**0.009**	**0.044**	**0.016**	1.000
SARC	203	**0.020**	0.145	0.139	1.000
SKCM	380	0.219	1.000	1.000	0.272
STAD	396	**0.019**	0.091	0.821	1.000
THCA	491	**0.020**	0.243	**0.042**	1.000
UVM	75	**<0.001**	**0.022**	**0.024**	1.000

Together, Table 1 and Fig 3 strongly suggest that the metastasis potential predicted by PLUS is highly consistent with the actual follow-up data for many different cancer types. This finding has three primary implications: (1) Gene expression at early stages can predict the propensity of patients to subsequently develop metastasis. (2) PLUS is the first prediction tool for cancer metastasis that works for a general set of cancer types, by harnessing the power of large-scale data integration. (3) The success of PLUS in predicting cancer metastasis potential further confirms that cancer metastasis is often under-detected, posing a threat to timely disease management.

### Functional mechanism of the metastasis predictive genes

A total of 191 metastasis-predictive genes were identified by PLUS that optimally predict metastasis potential in the TCGA pan-cancer data. A complete list of the 191 genes is provided in S3 Table. We first evaluated functional clusters of these genes by a pathway enrichment test against Msigdb canonical pathways and Gene Ontology [30]. The top 50 enriched pathways are mainly related to (1) responses to the oxidative stress such as hydroperoxide; (2) the regulation of calcium ion transport, and (3) responses to cytokines. More details are given in S4 Table. These pathways are known to be closely associated with cancer metastasis. In particular, hydrogen peroxide has been viewed as a “fertilizer” of inflammation, cancer metabolism and metastasis [31], and metastasis is the route for cancer cells to escape from the oxidative stress [32]. Moreover, the calcium ion is a ubiquitous second messenger that acts as crucial regulator of cell migration [33], and cytokines are central mediators in remodeling the local microenvironment to support the growth, survival, and invasion of primary tumors and enhance metastatic colonization [34].

We further investigated the correlations between all the individual genes and the PLUS predicted metastasis potential (see S5 Table) and selected the genes with significant positive correlations. Similar enrichment analysis demonstrated that the pathways positively associated with metastasis potential are non-surprisingly well-known metastasis-related pathways, and the most highly enriched genes are related to the immune system and inflammatory responses, extracellular matrix organization, and angiogenesis (see details on the enrichment results in S6 Table). This functional enrichment analysis presents partial evidence for the concordance of the PLUS-selected genes with the current body of literature. Below, we will examine whether these genes are truly potent in differentiating the metastasis potential of cancer cells using single-cell data.

### Validation of metastasis predictive genes in independent scRNA-seq datasets

To validate the metastasis-predictive genes selected by PLUS from TCGA pan-cancer data, we collected two scRNA-seq datasets of human breast and head and neck cancer. Both data sets contain cancer cells from cancer bulk tissue samples with different metastasis statuses (see details in the METHODS AND MATERIALS section). We first conducted cell clustering analysis on each dataset by using (1) general genes with high expression dispersion and (2) the 191 metastasis-predictive genes identified by PLUS. As reported in the original works [35–37], cancer cells from different patients possess strong inter-tumoral heterogeneity and tend to cluster together. Hence, the cancer cells in both breast and head and neck cancer data sets can be separated into two groups of primary cancer cells with high and low metastasis potential. A silhouette coefficient [38] was applied to determine whether cells of different metastasis potential are closer together in certain cell clustering results (see details in the METHODS AND MATERIALS section). Specifically, a larger silhouette coefficient value indicates that cells tend to be clustered together if they have similar metastasis potentials.

**Fig 4:**
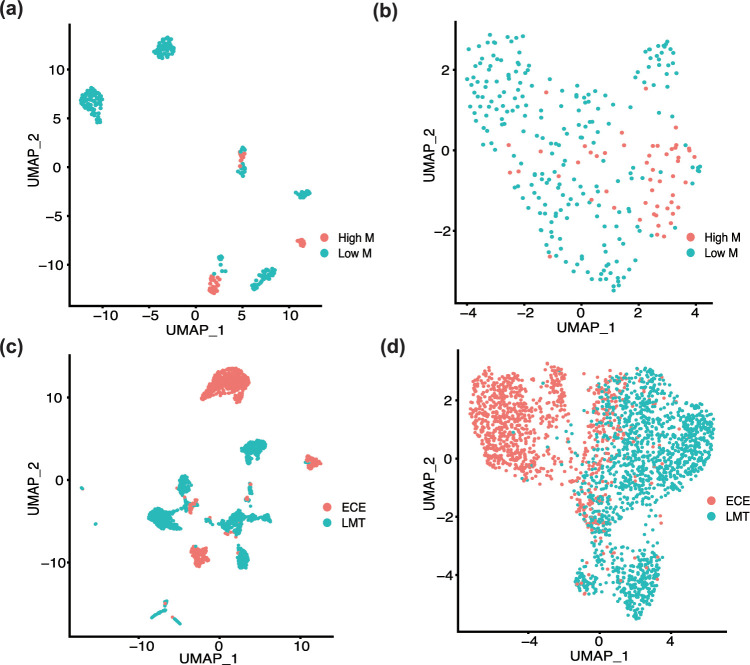
Functional validation of the metastasis-predictive genes inferred by PLUS from TCGA data. (a-b) Cell clusters of the cancer cells with high (High_M, red) and low (Low_M, blue) metastatic potential obtained by using all genes (a, silhouette coefficient = 0.07) and the PLUS selected metastasis predictive genes (b, silhouette coefficient = 0.36) in the scRNA-seq dataset for breast cancer (GSE75688). (c-d) Cell clusters of cancer cells from cancer tissues with (ECE, red) and without (LMT, blue) extracapsular extension obtained by using all genes (c, silhouette coefficient = 0.1) and the PLUS-selected metastasis-predictive genes (d, silhouette coefficient = 0.3) in the scRNA-seq dataset for head and neck cancer (GSE103322).

In the scRNA-seq data for breast cancer (GSE75688), the cancer cells were collected from three cancer tissues with high metastasis potential and seven tissues with low metastasis potential, determined by the number of lymph node metastases. Our analysis gave silhouette coefficients of 0.07 and 0.36 for the cells with high metastasis potential in the formed clusters when using all genes (see Fig 4A) and the PLUS-selected metastasis predicative genes (see Fig 4B), respectively. In the scRNA-seq dataset for head and neck cancer (GSE103322), cancer cells were collected from 6 patients with pathologically detected extracapsular extension, a significant indicator of a metastasis event at the primary site [39], and 14 patients without extracapsular extension. Cell clusters inferred by using all genes form distinct patient-specific groups (see Fig 4C), which does not show a strong dependency on the extracapsular extension event. In contrast, the cell clusters inferred from the PLUS metastasis-predictive genes clearly form two groups of cells, one from extracapsular extension cancer and one group of cells from the 14 cancer samples with lower metastasis potential (see Fig 4D). The average silhouette coefficients for the cells from extracapsular extension cancer are 0.1 and 0.3 in the cell clusters obtained by using all genes and the PLUS metastasis predictive genes, respectively. In addition to the performance of cell clustering analysis, we observed that TCGA-derived metastasis-predictive genes are significantly enriched by metastasis-potential-associated genes (24/147, *p* = 0.02 and 28/162, *p* = 0.0059), compared with background result (3037/31656 and 1977/21030) for the breast and head and neck cancer data, respectively.

Our analysis of independent scRNA-seq data sets clearly demonstrates that the 191 metastasis-predictive genes derived by PLUS from TCGA pan-cancer data are relevant to metastasis. Details regarding the analysis approaches applied to the scRNA-seq data are provided in the METHODS AND MATERIALS section.

### Robustness analysis

To evaluate the robustness of PLUS on the TCGA pan-cancer data, we intentionally removed the data from one cancer type at each time, and then ran PLUS using only the remaining samples. We examined the robustness of PLUS based on the overlap of the selected genes, as well as the correlations of the predicted metastasis potential on the M0 samples. In S1 Fig, we showed the number of overlapping genes and correlations of predicted metastasis potential, for any two PLUS predictions made with two different cancer type data removed. We also included the prediction with no samples removed (labeled as “ALL”). On the left panel, we observed that the correlation between any two predicted metastasis potential is consistently high, with a minimum correlation of 0.43, and a median of 0.90. For the overlapping genes, the minimum number of overlapping genes is 34, with a median of 116, for any pair of predictions. Note that PLUS implements a sparsity assumption on gene selection using *L*_1_ penalty, which may make the gene selection less stable. It is well known that sparsity and stability are at the odds of each other, especially when there is strong feature collinearity in the data [40], which may be the case for our gene expression data. Interestingly, six genes, including *ALS2*, *DAPL1*, *HS6ST1*, *IGFBP2*, *MGC12982*, *PPIAL4C*, are selected in all predictions, i.e., when no samples is removed, or one cancer type data is removed. The six genes are highly potential to be indicators of metastasis potential given their robustness, though they certainly warrant further experimental validation.

## Discussion and conclusions

Metastasis is the major cause of cancer-related deaths, and evaluations of metastasis risk are essential for tailored treatment of cancer patients. Existing computational tools for predicting the cancer metastasis potential fall under two categories: 1) methods that build a classifier using the clinical metastasis diagnoses as responses and 2) methods that evaluate the behavior of gene features found to be significantly associated with metastasis-related survival outcomes. Such predictors exist in many even for the same cancer type; however, selected gene features rarely overlap, not to mention the little consistency of metastasis predictor genes among different cancer types. Thus, there is an urgent need for a powerful tool to characterize the cancer metastasis potential and to delineate the important gene features of cancer metastasis that is applicable across a wide span of cancer types.

Traditional classification methods for predicting cancer metastasis overlook an important fact in cancer metastasis diagnosis: while it is easy to confirm metastasis events with detected metastatic cancer cells in lymph nodes or distant locations, it is much more challenging to confirm non-metastasis events. Disseminating cancer cells may undergo hibernation, temporarily causing few or no complications in the patients, and clinical procedures are often not sufficiently accurate to capture ongoing events. In both cases, we see that cancer metastasis events tend to be under-diagnosed. Comparing the initial metastasis diagnosis and follow-up metastasis occurrence in TCGA pan-cancer clinical data confirmed this unfortunate finding: despite an initial non-metastatic diagnosis, many patients of various cancer types develops metastasis in the following years (see Fig 1B). A good classifier for metastasis should be designed to account for this under-diagnosis issue. However, finding prognostic markers from survival data by treating metastasis as a censored event may not reveal the genes with true biological and functional relevance to metastasis.

Our proposed PLUS algorithm builds on the framework of PU learning by considering patients with metastasis diagnosis as positive instances and the remainder as unlabeled instances, meaning they are either metastatic or non-metastatic. Under this framework, the selected genes become truly relevant to the biology of metastasis. Indeed, the classifier given by PLUS rendered concordance between the predicted cancer metastasis and observed metastasis survival outcomes in the follow-up data for almost all cancer types considered. The selected genes were found to perform functions consistent with experimental research findings and are capable of clustering the single cells based on their levels of metastasis potential. PLUS fully exploits the power of big data by training on ~7,000 patients samples, where only a very small portion are diagnosed as metastatic samples. The superiority of PLUS over other methods lies in its tailored designed that overcomes the high dimensionality of gene features, the unbalancedness issue in instance allocation (more non-metastatic than metastatic diagnoses), and the possible unbalancedness in the underlying population distribution (unknown population prevalence of metastasis), which fully recapitulates the case of cancer metastasis. The computational tool designed and insights gained from this research will prove useful to the diagnosis and treatment of clinical metastatic disease.

Notably, while different cancer types and subtypes may have different metastasis mechanisms, the successful application of PLUS to pan-cancer data demonstrates its power to identify common hallmarks for early metastasis prediction across cancer types, confirming the accuracy, reliability, and robustness of this model. In addition, the gene markers identified by PLUS are related to early metastasis events, including a series of actions for invading cancer cells to overcome stromal barriers, survive in the circulation system, and settle and colonize at a distant metastasis site, which have been revealed as common metastasis hallmarks for diverse cancer types. In fact, researchers have been harnessing the power of big data by integrating the omics data of multiple cancer types to find biomarkers that underlie a common pathway of oncogenesis and particularly the EMT process [41,42]. As a result, a pan-cancer EMT signature gene has been discovered that is independent of cancer types [43]. These findings suggest the rationality of applying PLUS to pan-cancer data.

## Methods and materials

### Model setup

Let $x\in R^{p},y\in R$ be a *p*-dimensional covariate and binary response variable. To model the probability of observing an event *y* conditioning on covariates *x*, logistic regression is commonly used to estimate the probability *Pr*(*y* = 1|*x*) when both positive outcomes *y* = 1 and negative outcomes *y* = 0 occur in the observation. In the PU setting, however, only positive labeled instances are observed, while the labels for the remaining instances are unknown, or too noisy, as in the case of cancer metastasis diagnosis. In other words, we denote the observed outcome by *z*, where *z* = 1 represents positively labeled instances, and *z* = 0 represents unlabeled instances. For a subject *i* with *z*_*i*_ = 1, it clearly follows that *y*_*i*_ = 1. However, if *z*_*i*_ = 0, then either *y*_*i*_ = 1 or *y*_*i*_ = 0. The main purpose of PU-learning is to estimate *Pr*(*y*_*i*_ = 1|*x*_*i*_) from observed data tuples (*x*,*z*). Direct application of logistic regression in which *z* is treated as the response is severely biased. Because only part of *y* is observed, the PU problem can be viewed as a missing data problem, and one commonly used method for missing data problems is the EM algorithm [24]. In the following sections, we will first introduce the existing EM algorithm designed for the PU problem and then propose our PLUS algorithm, which is more tailored for the unbalancedness and sparsity issues in cancer metastasis prediction.

### Case-control framework

We adopt the case-control framework proposed by Wald et al. [24], i.e., the positive instances are sampled from one distribution, deemed as “cases”, while the negative instances are sampled from the other one, deemed as “controls”. It is based on two reasonable assumptions:

**Assumption 1**: Positive instances are completely randomly selected from the positive population. In other words, whether an instance is observed as positive is regardless of its covariates *x*, that is,

Pr(z=1|x,y=1,s=1)=Pr(z=1|y=1,s=1);

**Assumption 2**: The unlabeled instances are a random sampling from the population, that is:

Pr(y=1|x,z=0,s=1)=Pr(y=1|x)

Here, *s* = 1 indicates that an instance is in the sample, which is always the case when we are working with the sample. The observed likelihood under this case-control sampling scheme is

$$\begin{array}{l} L^{obs}(\theta |x,z,s=1)=\prod_{i}P_{\theta }(z_{i}|s_{i}=1,x_{i})\\ \;={\prod_{i}P_{\theta }(z_{i}=1|s_{i}=1,x_{i})}^{z_{i}}{\left(1-P_{\theta }(z_{i}=1|s_{i}=1,x_{i})\right)}^{1-z_{i}}, \end{array}$$ (1)

and the full likelihood is

$$\begin{array}{l} L^{full}(\theta |x,y,z,s=1)=\prod_{i}P_{\theta }({y_{i},z}_{i}|s_{i}=1,x_{i})\\ \;\propto \prod_{i}P_{\theta }(y_{i}=1|s_{i}=1,x_{i}{)}^{y_{i}}P_{\theta }(y_{i}=0|s_{i}=1,x_{i}{)}^{{1-y}_{i}}. \end{array}$$ (2)

Direct optimization with respect to the observed likelihood function is difficult; thus, an EM procedure is introduced to accomplish the optimization according to the expectation of the observed likelihood.

E-step:

Given the estimated model parameter *θ*^(*k*)^ from the *k*^*th*^ iteration, the conditional expectation of full log-likelihood is thus

$$\begin{array}{l} Q(\theta |\theta^{\left(k\right)})=E[l^{full}(\theta |x,y,z,s=1)|x,z,s=1,\theta^{\left(k\right)}]\\ \;=\sum_{i}\left\{E[y_{i}|z_{i},x_{i},s_{i}=1,\theta^{\left(k\right)}]logf_{\theta }^{*}(x_{i})+(1-E[y_{i}|z_{i},x_{i},s_{i}=1,\theta^{\left(k\right)}])log(1-f_{\theta }^{*}(x_{i}))\right\}, \end{array}$$

where $f_{\theta }^{*}(x_{i})=P_{\theta }(y=1|x,s=1)$. We are also aware of

$$E[y_{i}|z_{i},x_{i},s_{i}=1,\theta^{\left(k\right)}]=P_{\theta^{\left(k\right)}}(y_{i}=1|z_{i},x_{i},s_{i}=1)=f_{\theta^{\left(k\right)}}{\left(x_{i}\right)}^{\left(1-z_{i}\right)},$$

where $f_{\theta^{\left(k\right)}}(x)=P_{\theta^{\left(k\right)}}(y_{i}=1|x_{i})$. This expression holds because when *z* = 1, the outcome *y* can only equal to 1, and when *z* = 0, we know from Assumption 2 that *Pr*(*y* = 1|*x*, *z* = 0, *s* = 1) = *Pr*(*y* = 1|*x*).

M-step:

In the M-step, we maximize the expectation of full log-likelihood described in the E-step with a penalty term *λJ*(*θ*) to account for the sparsity

$$\theta^{\left(k+1\right)}=\arg\;{max}_{\theta }\;Q(\theta |\theta^{\left(k\right)})+\lambda J(\theta ),$$ (3)

where *λ* is a penalty coefficient, and *J*(·) is a proper regularization function. Here, we adopt the *L*_1_ norm to select informative variables. The penalized likelihood method based on EM was implemented in a similar manner as PUlasso [26].

After the M-step, we obtained *θ*^(*k*+1)^ as well as $f_{\theta^{\left(k+1\right)}}^{*}$, and to obtain $f_{\theta^{\left(k+1\right)}}$ for the next E-step, we derive the connection between these two terms (see Supplementary Methods for detailed derivations):

$$f_{\theta }(x)=\frac{\left(c-1)f_{\theta }^{*}(x\right)}{c-f_{\theta }^{*}(x)},$$ (4)

where

$$c=\frac{Pr(y=1|s=1)}{Pr(z=1|s=1)}.$$

*Pr*(*z* = 1|*s* = 1) is directly observed in the sample, but *Pr*(*y* = 1|*s* = 1) requires knowledge of the population prevalence *π* = *P*(*y* = 1). However, this parameter is unknown in our case, as we do not have prior information on the population prevalence of metastasis, which is indeed what we are seeking. A randomly assigned population prevalence may work as well when the two classes in the population are clearly separated and have a balanced presence; however, as we will see in the simulation data, this approach severely impacts PUlasso performance when the true prevalence is close to 0 or 1, or, in other words, when the population allocation is unbalanced.

### PLUS framework

Even with the penalized likelihood estimation to enable feature selection, using such a framework similar to PUlasso for predicting cancer metastasis potential is not applicable for two reasons: 1) the observed unbalancedness, in which there are fewer positive samples (metastatic diagnosis) than unlabeled samples (non-metastatic diagnosis), and 2) unknown population prevalence, meaning that there is no prior knowledge on how many patients are metastatic in the population. Both challenges may significantly impact the performance of the case-control framework.

To address the challenges in existing algorithms, the proposed PLUS algorithm is particularly tailored to deal with potential unbalancedness in both observation and population allocation, along with a sparse data structure. Sparsity is solved by adopting a variable selection procedure, which can be naturally embedded in the EM structure with the LASSO penalty in the M-step. Unbalancedness can occur at 1) population level or 2) observation level as follows. 1) The population prevalence is extreme, that is, *π* is either close to 0 or close to 1, or 2) the number of observed positives is outnumbered by the unlabeled instances. Both types of unbalancedness, along with the PU setting, makes this problem even more complicated.

We rewrite *c* in Eq (4) as

$$c=1+\mathrm{Pr}\left(y=1|z=0,s=1\right)\frac{\mathrm{Pr}\left(z=0|s=1\right)}{\mathrm{Pr}\left(z=1|s=1\right)},$$ (5)

where both types of unbalancedness are explicitly included. The population unbalancedness is expressed by *Pr*(*y* = 1|*z* = 0, *s* = 1), since *Pr*(*y* = 1|*z* =0, *s* = 1) = *Pr*(*y* = 1) under Assumption 2. Meanwhile, $\frac{Pr(z=0|s=1)}{Pr(z=1|s=1)}$ is a measure for observation unbalancedness. Clearly, when the positive prevalence is high or the unlabeled instances outnumber the positive instances, *c* will be a relatively large number. From S2 Fig, we find that the larger the *c*, the little difference between $f_{\theta }^{*}$ and *f*_*θ*_. Consequently, when these unbalanced scenarios occur, the traditional EM-based algorithm approach is prone to perform little correction on the $f_{\theta }^{*}$. In practice, this behavior causes PUlasso to fail in unbalanced situations.

According to this observation, we propose a new way to transform $f_{\theta }^{*}$ to *f*_*θ*_, which does not require a knowledge of the population prevalence *P*(*y* = 1) and also works for unbalanced scenarios. Unlike the EM algorithm, in which $f_{\theta }^{*}(x)$ is always smaller than *f*_*θ*_(*x*), our transformation adopts a bipolar function such that extreme estimated probabilities will become more extreme. Here, we choose a sigmoid function:

$$f_{\theta }(x)=\frac{1}{1+e^{-\alpha g(f_{\theta }^{*}(x)-p_{0})}},$$ (6)

where *p*_0_ is the anchor probability. Based on this sigmoid function, any $f_{\theta }^{*}(x)$ larger than *p*_0_ will be projected from 0.5 to 1 or 0 to 0.5 for those smaller than *p*_0_. *p*_0_ is determined by the *q*_0_-th percentile of the estimated probability for the positive cases, or *E*(*y*|*x*, *z* = 1, *s* = 1). Here, we use the predicted probability of the positive samples to help distinguishing the unlabeled instances. PLUS is not sensitive to the choice of *q*_0_ if the rank of probability is applied (see S3 Fig). *g*(·) is a function that linearly maps $f_{\theta }^{*}(x)-p_{0}$ to an arbitrary symmetric domain of the sigmoid function, for example [−1, 1], calibrated at 0. *α* is a scale parameter that determines the magnitude of transformation. In practice, this parameter parimarily determines the speed of convergence. We suggest choosing a value between 5 to 10 if the domain is [−1, 1]. In S2 Fig, we show a direct comparison of the sigmoid transformation and the EM transformation.

**Fig 5:**
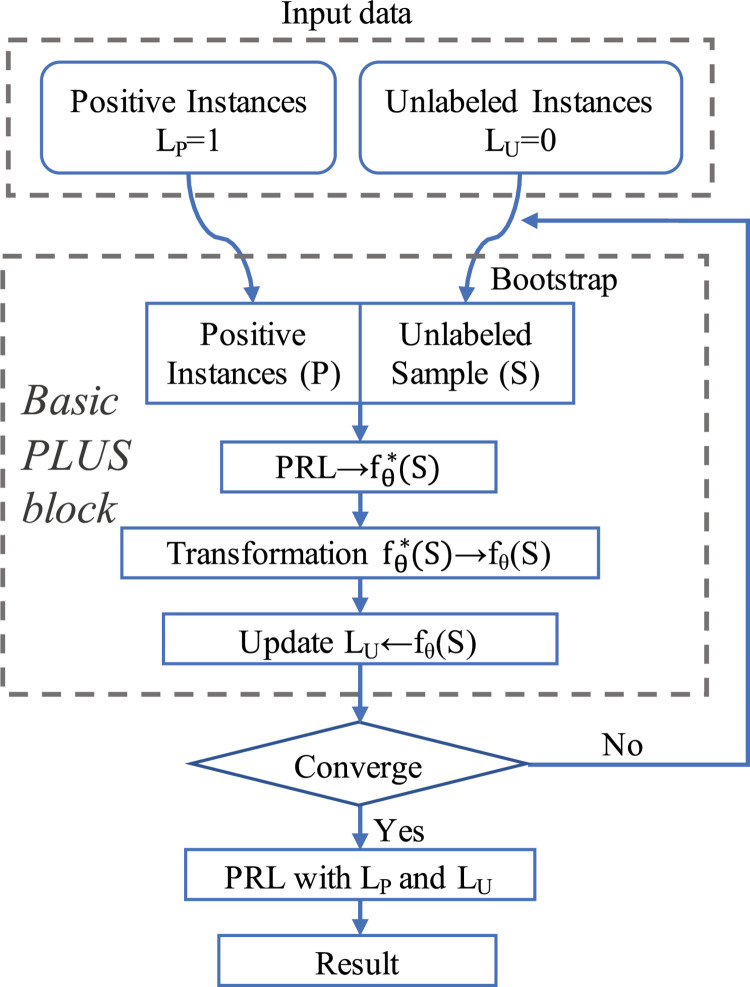
A schematic plot of the PLUS algorithm.

At each iteration, we 1) randomly sample the same number of observed positive instances from the unlabeled set with replacement and 2) conduct a one-step EM calculation, but only use only the new transformation function (6). In this manner, we can handle observation unbalancedness by maintaining a reasonably high ratio at each step. Then we 3) update the estimated probability for each unlabeled instance. Repeat step 1–3 until the estimated probabilities are stabilized. We take advantage of this bootstrap scheme to reduce the noise and increase the robustness. The details of the algorithm, as well as a flowchart, are given in Box 1: Algorithm 1 (PLUS) and Fig 5.

Note that, theoretically, the penalized logistic regression adopted in each iteration requires binary outcomes, while *E*[*y*_*i*_|*z*_*i*_ =0, *x*_*i*_, *s*_*i*_ = 1, *θ*^(*k*)^] typically lie between 0 and 1 and are not binary. However, most logistic procedures are currently able to handle non-integer responses, or we can work on augmented dataset as long as weights can be incorporated [24]. Thus, adopting *E*[*y*_*i*_|*z*_*i*_ =0, *x*_*i*_, *s*_*i*_ = 1, *θ*^(*k*)^] as responses is computationally feasible.

**Box 1: Algorithm 1 (PLUS)***Input: X_M×N_(Covariate matrix), the indices and labels of positive instances P, and L_P_, its size N_0_, and parameters q_0_, α*.

$Output:\;f_{\theta^{final}}$

*Initialization: Labels for the unlabeled instances L_U_ = **0***

***While** stopping criteria are not met, **do***

    *1. Randomly sample *N*_0_ unlabeled instances with replacement, denoted as *S*, where *N*_0_ is the number of positive instances*.

    *2. Run a PLR based on all positive instances P and *S*. Record the estimated outcomes for P and S, which are $f_{\theta }^{*}(P)$ and $f_{\theta }^{*}(S)$*.

    *3. Calculate *p*_0_ based on the *q*_0_-th percentile of $f_{\theta }^{*}(P)$*.

    *4. Perform a sigmoid transformation on $f_{\theta }^{*}(S)$ by*

        $f_{\theta }(S)=\frac{1}{1+e^{-\alpha g(f_{\theta }^{*}(S)-p_{0})}}$

    *5. Update the corresponding labels for S by *f*_*θ*_(*S*)*:

      *L_U_[S]←f_θ_(S)*

***End***;

*Run a PLR based on L_P_ and the new L_U_, to yield the final estimation $f_{\theta^{final}}$, which tells the selected features, as well as the class probability, i.e., P(Y = 1|X)*.

### Simulation settings of synthetic data

To comprehensively assess the proposed method, we designed a series of simulation studies. Four different scenarios for the noise distribution were simulated, corresponding to a balanced population with two well-separated classes (a clear balanced scenario), a balanced population with two classes not well-separated (a noisy balance scenario), an unbalanced population with two well-separated classes (a clear unbalanced scenario), and an unbalanced population with two classes not well-separated (a noisy unbalanced scenario). Alterations to the population unbalancedness and separation are achieved by designing different propensity score functions as follows:

Clear Balanced Scenario: $logit(\mathrm{Pr}\left(Y=1\right))=2X_{1}+4X_{2}{(X}_{3}+5)-3\;\sin\left(X_{4}+X_{5}\right)-0.1X_{6}^{4}$

Noisy Balanced Scenario: $logit(\mathrm{Pr}\left(Y=1\right))=2X_{1}+4X_{2}X_{3}-3\;\sin\left(X_{4}+X_{5}\right)-0.1X_{6}^{4}$

Clear Unbalanced Scenario: $logit(\mathrm{Pr}\left(Y=1\right))=2X_{1}+8X_{2}X_{3}-3\;\sin\left(X_{4}+X_{5}\right)-{3(X_{6}-1)}^{4}$

Noisy Unbalanced Scenario: $logit(\mathrm{Pr}\left(Y=1\right))=2X_{1}+1.5X_{2}X_{3}-3\;\sin\left(X_{4}+X_{5}\right)-{\left(X_{6}-1\right)}^{4}$

The functions look similar, but their non-linear properties are distinguished. Here, we show distributions of *Pr*(*Y* = 1) for each of the scenario in S3 Fig. In addition, the direction of the population unbalancedness is also altered, causing the positive instances to be either the larger or smaller class.

We simulate the covariate matrix *X*_n×p_~*MVN*(0, *I*_*p*×*p*_), where the total number of covariates *p* can take is 100, 200, and 400 and the sample size *n* is 2000. For all environments, only six of the variables are relevant to the binary outcome *Y*. To generate PU instances, we randomly flip the true label 1 to 0, and the probability of a sample being flipped determines the observation balancedness. A higher flipping probability corresponds to a lower observation balancedness, with the sum of these two values equaling 1. The level of observation balancedness ranges from 0.2 to 0.5. Its impact is shown in Fig 2 on the *x*-axis.

### Benchmark methods and comparison

PLUS was compared with six state-of-the-arts PU learning or binary classification methods and one oracle reference method: (1) The **PUlasso** algorithm, which implements the EM algorithm for penalized likelihood estimation [26], and it utilizes a majorization-minimization framework to improve the stability of the EM-algorithm-based solution. The (2) **Ada-KNN**, (3) **Ada-Logit**, and (4) **Ada-SVM** methods all belong to a multi-method wrapper, called AdaSample [27]. AdaSample utilizes an adaptive sampling procedure to estimate the class mislabeling probability and to reduce the risk of selecting mislabeled instances. It is presented as a wrapper that can integrate support vector machine (SVM), k-nearest neighbor (KNN), logistic regression (logit), linear discriminant analysis (LDA), and feature weighted KNN [2]. (5) **XGBoost** is a state-of-the-art decision-tree-based classification algorithm that uses a gradient boosting framework [44]. (6) The **random forest** is a classification algorithm consisting of many decisions trees, which uses bagging and feature randomness when building each individual tree in an attempt to create an uncorrelated forest of trees whose combined prediction is more accurate than that of any individual tree [45]. (7) **PRL** places an L1 penalty on the logistic regression coefficients to enable variable selection. Notably, the PLR method was applied to the synthetic data using the true underlying label for method validation. For real data, PLR was also applied as a baseline method with the observed labels treated as true, which leads to an underestimated metastasis potential. Of all the methods, the Ada- methods, like most of the PU-learning methods, are incapable of variable selection. For each simulation setting, we conducted 100 repetitions. In each repetition, a predictive model is trained by each method, which generates an ROC curve with false and true positive rates at the *x*- and *y*- axis and the AUC. The average AUC among the 100 repetitions is used to evaluate the prediction performance for each method and simulation setting.

### Data analyzed in this study

#### TCGA transcriptomics data

We retrieved the RNA-seq data from the PanCanAtlas [46]. The retrieved data includes the expression profiles of 20,531 genes in 10,332 samples from 33 cancer types. We combined COAD and READ into one cancer type called COADREAD. Among all cancer types, we extracted the 20 cancer types with at least one sample that was initially diagnosed as metastatic, with a total sample size of 7,467. Among these samples, 553 were confirmed to be metastatic. The detailed cancer types and corresponding frequencies of initial metastasis diagnosis, are listed in S1 Table. We first applied EB++ to remove the batch effect introduced by the different cancer datasets, as suggested by PanCanAtlas. EB++ is a recently developed batched effect removal method for TCGA pan-cancer data analysis that can adjust for sequencer platform differences (https://bioinformatics.mdanderson.org/BatchEffectsViewer/). Specifically, the UNC GA- and BCCA GAII-sequenced samples were separately adjusted to the UNC HiSeq data.

#### TCGA clinical information

Baseline and follow-up clinical information for all cancer samples was retrieved from the Genomic Data Commons Data Portal. Baseline diagnosis of a distant metastasis event was retrieved from the baseline clinical data, based on whether the patient is diagnosed as stage M1 in the TNM stage information. Specifically, a patient is determined to be diagnosed with metastasis if (1) the patient is in the M1 stage according to at least one of the “ajcc_metastasis_pathologic_pm”, “ajcc_metastasis_clinical_cm”, “clinical_M”, and “pathologic_M” criteria in the TCGA clinical information or (2) there exists direct evidence of metastasis under one of the following terms: “metastatic_dx_confirmed_by”, “metastatic_dx_confirmed_by_other”, “metastatic_tumor_site”, “metastasis_site”, “metastatic_site_other”, “metastasis_site_other”, “metastatic_tumor_indicator”, “metastatic_disease_confirmed”, “metastatic_site”, and “other_metastatic_site”. Based on these criteria, 553 samples were confirmed to have a metastasis diagnosis while the remainder were treated as unlabeled samples.

To evaluate the accuracy of the predicted metastasis potential, we utilized the available TCGA follow-up data and collected PFS data. We specifically defined events in PFS as a patient having a new tumor event, whether this event was a progression of disease, local recurrence, distant metastasis, new primary tumor at any sites, or deceased with the cancer and no new tumor event, including cases with a new tumor event whose type is N/A, based on which the PFS specific to metastasis events can be computed. The unit of progression-free interval time is days. For the progression events, we collected either new_tumor_event_dx_days_to or death_days_to, whichever was applicable, and for the censored cases, we collected either last_contact_days_to or death_days_to, whichever was applicable. These collection criteria are in accordance with the existing literature [47].

#### scRNA-seq datasets

Two scRNA-seq data sets for human breast cancer (GSE75688) and head and neck cancer (*GSE103322*) were retrieved from the Gene Expression Omnibus database [35–37]. The datasets were selected based on the presence of cancer cells of varied metastasis status in the original bulk tissue or bulk cell samples. All data were downloaded as the counts or TPM profiles used in the original work. Cell labels generated in the original work were directly utilized. Specifically, GSE75688 includes data for 281 cancer cells from 10 cancer tissues, of which 3 have a high metastasis potential defined by metastasis to more than 2 lymph nodes and 7 have a low metastasis potential. *GSE103322 contains data for 1040 cancer cells from 6 primary* head and neck cancer *tissues with* extracapsular extension, a significant indicator of a metastasis event at a primary sit*e*, *and 1468 cancer cells from 14 primary* cancer *tissues without* extracapsular extension. Notably, the cancer cells from each scRNA-seq dataset can be classified as having high or low metastasis potential by the provided pathological or phenotypic information.

### Analysis of scRNA-seq data

Both GSE75688 and GSE103322 were collected by using C1-Fluidigm or C1-SMART-seq protocol, and the sequencing saturation for both datasets are high. We selected only the malignant cells based on the cell labels provided in the original works. Seurat 3.0 was utilized for basic data processing [48]. All the analyzed cells have at least 1000 UMI measured. Cell clustering analysis was conducted via Seurat 3.0 with default parameters. Specifically, the cell clusters inferred from all genes were based on all genes with significant dispersion detected by Seurat, and the clustering inferred from the metastasis-predictive genes was based on the intersection of the metastasis-predictive genes identified by PLUS from the TCGA dataset and the significantly varied genes in each scRNA-seq dataset. The silhouette width *s*(*i*) of each cell and the silhouette coefficient defined below were utilized to determine whether the cells from the tissues with higher metastasis potential were more closely clustered when the metastatic-predictive genes were used. We note that there are only two oracle cell clusters, namely, cells of high and low metastasis potential, in each dataset.

$$a(i|i\in C_{m})=\frac{1}{|C_{m}|-1}\sum_{j\in C_{m},i\neq j}d(i,j),b(i|i\in C_{m})=\frac{1}{\left|C_{nm}\right|}\sum_{j\in C_{nm}}d(i,j)$$

$$s(i|i\in C_{m})=\frac{b(i)-a(i)}{max\{a(i),b(i)\}}$$

$$Silhouette\;coefficient\;(C_{m})=mean(s(i|i\in C_{m})),$$

where *C*_*m*_ and *C*_*nm*_ represent cells with high and low metastasis potential, *d*(*i*,*j*) is the distance between cells *i* and *j* in the dimension-reduced space, and *s*(*i*) is the silhouette width of the cell *i*. Notably, only the cells in *C*_*m*_ are utilized to compute the silhouette width and silhouette coefficient. The rationale here is that the silhouette coefficient can validate the use of the predicted metastasis-predictive genes and can better cluster the cells of high metastasis potential in independent scRNA-seq data. Here, 2D UMAP-based dimension reduction was utilized for the results visualization and computation of the distance *d*(*i*,*j*). The average *s*(*i*) values of the cells with high metastasis potential derived from the cell clusters inferred by using all genes or metastasis-predictive genes were compared, where a higher average silhouette width suggests a better clustering of the cells with high metastasis potential.

In addition to the silhouette width, we also assessed whether the PLUS-selected metastatic-predictive genes are more enriched by genes that are significantly associated with the metastasis status in each scRNA-seq dataset. For each gene, the association between the single cells’ gene expression and their metastasis potential, which is defined as the metastasis potential of the patients where the cells were derived from, was tested by a Student’s t-distribution-based test of their Pearson correlation, with p<0.01 as the significance cutoff. Genes with significant positive association with the metastasis potential are called metastasis Enrichment of the scRNA-seq data-derived metastasis-associated genes in the TCGA data-derived metastatic-predictive genes was tested by a hypergeometric test.

## Supporting information

S1 Table TCGA cancer type, their full names, and initial metastasis diagnosis. (CSV)

S2 Table TCGA sample and clinical information, and predicted metastasis potential by the four methods. (CSV)

S3 Table The 191 metastasis predictive genes inferred by PLUS from TCGA pan-cancer data. (CSV)

S4 Table The top enriched pathways using the 191 metastasis predictive genes. (CSV)

S5 Table The Spearman correlation between expression of all genes and PLUS predicted metastasis potential. (CSV)

S6 Table The enrichment analysis using genes positively correlated with the PLUS predicted metastasis potential. (CSV)

S1 Fig The number of overlapping genes (left panel) and correlations of predicted metastasis potential (right panel), for any two PLUS predictions made with two different cancer type data removed. (EPS)

S2 Fig Transformation from f* to *f*. Solid lines: EM transformation; Dashed line: Proposed sigmoid transformation with *p*_0_ = 0.5. (TIF)

S3 Fig Examples of the level of population unbalancedness and level of separation of two classes. The x-axis is the probability of a positive label and the y-axis is frequency. (a) Noisy Balance represents the case of the positive and negative labels are balanced, and the two classes are less separable. (b) Noisy Unbalanced represents the case of the positive and negative labels are unbalanced, and the two classes are less separable. (c) Clear Balanced represents the case of the positive and negative labels are balanced, and the two classes are separable. (d) Clear Unbalanced represents the case of the positive and negative labels are unbalanced, and the two classes are separable.
(TIF)

S1 Text It contains the supplementary methods and references. (DOCX)
