# Supplementary figures and images for: PLUS: Predicting cancer metastasis potential based on positive and unlabeled learning
Source: PLoS Comput Biol. 2022 Mar 29;18(3):e1009956. doi: 10.1371/journal.pcbi.1009956 (PMC8992993; doi:10.1371/journal.pcbi.1009956)

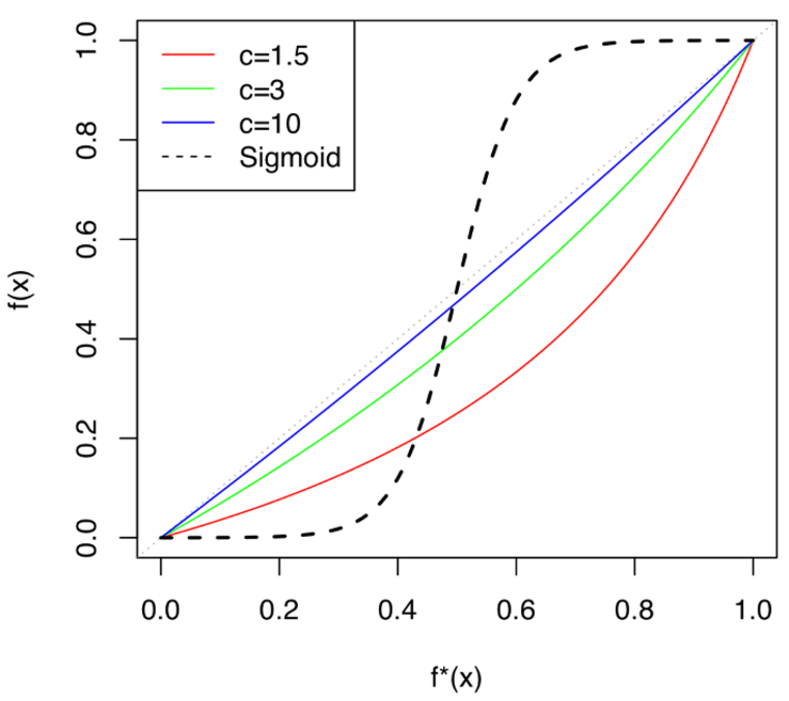

Supplement: S2 Fig — (TIF) [file pcbi.1009956.s008.tif]

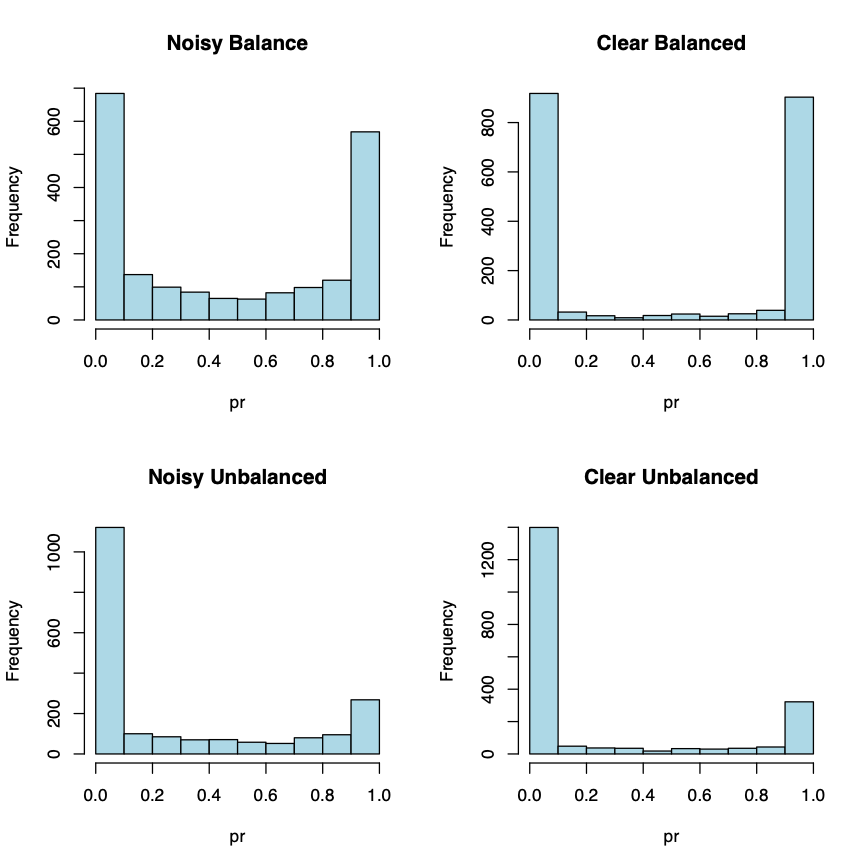

Supplement: S3 Fig — The x-axis is the probability of a positive label and the y-axis is frequency. (a) Noisy Balance represents the case of the positive and negative labels are balanced, and the two classes are less separable. (b) Noisy Unbalanced represents the case of the positive and negative labels are unbalanced, and the two classes are less separable. (c) Clear Balanced represents the case of the positive and negative labels are balanced, and the two classes are separable. (d) Clear Unbalanced represents the case of the positive and negative labels are unbalanced, and the two classes are separable. (TIF) [file pcbi.1009956.s009.tif]
