# Supplementary material for: PLUS: Predicting cancer metastasis potential based on positive and unlabeled learning
Source: PLoS Comput Biol. 2022 Mar 29;18(3):e1009956. doi: 10.1371/journal.pcbi.1009956 (PMC8992993; doi:10.1371/journal.pcbi.1009956)
Supplement: S1 Text — (DOCX) [file pcbi.1009956.s010.docx]

Details on the simulation procedures used to generate Figure 2 and the performance comparisons for varying number of informative features not shown in the main text, and analysis procedures of the TCGA pan-cancer data and single cell RNA-Seq data could be found in the GitHub page of PLUS: <https://github.com/xiaoyulu95/PLUS>.

**SUPPLEMENTARY METHODS**

**1. Related work on PU learning**

Positive and unlabeled learning, or positive-unlabeled (PU) learning, refers to the binary classification problem where only positive labels are observed, and the rest are unlabeled. Since unlabeled part of data consists of both positive and negative instances, naively treating them as negative and performing a standard classification learning algorithm will underestimate the probability of being positive [1, 2]. Without providing negative instances in the training set, however, will prevent the direct use of well-developed supervised classification methods. To break through this dilemma, dozens of PU learning algorithms have been proposed in the past two decades.

One way to bypass the lack of negative instances is to disregard the unlabeled part and only learn from positive instances. Given this underlying idea is similar to one-class classification problem, which is designed do classification when the negative instances are absent, poorly sampled, or not well defined [3], many existing one-class learning algorithms could be easily formulated to PU learning [4], such as Positive Naive Bayesian (PNB) [5, 6], one-class SVM [7-9], and one-class KNN [10]. However, since unlabeled instances are not used during the training step, it may not be competitive to those algorithms which could effectively utilize information from both positive and unlabeled instances.

To better utilize the unlabeled instances, one class of algorithms adopt a heuristic two-step strategy[11-15]. In the first step, instances that are likely to have negative labels are identified by certain similarity, or distance metrics. In the second step, a classifier is developed based on the positive instances, quasi-negative instances detected in the first step, and remaining unlabeled instances. Alternatively, another branch of methods let positive and unlabeled instances share different weights in the loss function and/or classification model to account for the asymmetric nature of PU learning problem. Many existing classification algorithms that are able to incorporate weights have been studied, such as naive Bayes [16], biased SVM [14], and biased logistic model [17]. One potential drawback of the aforementioned methods is they either explicitly or implicitly rely on the assumption that data are generated from a mixture model [16]. Hence, they are more appropriate for deterministic scheme but not probabilistic scheme, following the nomenclature proposed by Song et al. [18].

Meanwhile, a more theoretical viewpoint of PU learning has been developed by putting it into a case-control framework [1], given the fact that the way of sampling is not the same for positive set and unlabeled set. The main disadvantage of this method is we need to know the population prevalence, which is almost impossible in practical application, otherwise it is not identifiable with observed positive and unlabeled instances [1].

Recently, researchers incorporate bagging [19] idea into the PU learning to generate final classifier by ensembling multiple PU classifiers estimated from bootstrap sampling [20-23]. This approach takes advantage of bagging feature to reduce noise from modeling directly with unlabeled instances and obtain more stable predictions. Rather than using unanimous probability to sample, AdaSample [24], a more boosting-like algorithm, applies different sampling probability at each step, and the probability is calculated from PU model estimated in the previous iteration. The performance of the adopted PU classifier for the bootstrap sample is of great importance to the success of this set of methods.

**2. The derivation of equation (4) in the main text**

By Bayesian formula, we have following relationship:

$$Pr\left( s=1|y=1,x \right)=Pr\left( s=1|y=1 \right)\frac{Pr\left( y=1|s=1 \right)}{Pr\left( y=1 \right)}Pr\left( s=1 \right)$$

$Pr\left( y=1|s=1 \right)=Pr\left( y=1|z=1,s=1 \right)Pr\left( z=1|s=1 \right)+Pr\left( y=1|z=0,s=1 \right)Pr\left( z=0|s=1 \right)$

= $Pr\left( z=1|s=1 \right)+Pr\left( y=1|z=0,s=1 \right)Pr\left( z=0|s=1 \right)$

$$\Longrightarrow Pr\left( s=1|y=1,x \right)=\left[ \frac{Pr\left( z=1|s=1 \right)}{Pr\left( y=1 \right)}+Pr\left( z=0|s=1 \right) \right]Pr\left( s=1 \right)$$

$$Pr\left( s=1|y=0,x \right)=Pr\left( s=1|y=0 \right)=\frac{Pr\left( z=0|s=1 \right)}{Pr\left( y=0 \right)}Pr\left( s=1 \right)$$

$$Pr\left( y=0|s=1 \right)=Pr\left( y=0|z=1,s=1 \right)Pr\left( z=1|s=1 \right)+Pr\left( y=0|z=0,s=1 \right)Pr\left( z=0|s=1 \right)$$

$$=Pr\left( y=0 \right)Pr\left( z=0|s=1 \right)$$

$$\Longrightarrow Pr\left( s=1|y=0,x \right)=Pr\left( z=0|s=1 \right)Pr\left( s=1 \right)$$

Plug previous relationships into the following equation, we obtain

$$f_{\theta}^{*}\left( x \right)=P_{\theta}(y=1|x,s=1)$$

$$=\frac{Pr\left( s=1|y=1,x \right)P_{\theta}(y=1|x)}{Pr\left( s=1|y=1,x \right)P_{\theta}\left( y=1 | x \right)+Pr\left( s=1|y=0,x \right)P_{\theta}(y=0|x)}$$

$$=\frac{\frac{Pr\left( y=1|s=1 \right)}{Pr\left( y=1 \right)}f_{\theta}\left( x \right)}{\frac{Pr\left( z=1|s=1 \right)}{Pr\left( y=1 \right)}f_{\theta}\left( x \right)+Pr\left( z=0|s=1 \right)}$$

$$=\frac{\frac{Pr\left( y=1|s=1 \right)}{Pr\left( z=1|s=1 \right)}f_{\theta}\left( x \right)}{f_{\theta}\left( x \right)+\frac{Pr\left( z=0|s=1 \right)Pr\left( y=1 \right)}{Pr\left( z=1|s=1 \right)}}$$

$$=\frac{cf_{\theta}\left( x \right)}{f_{\theta}\left( x \right)+c-1}$$

or equivalently,

$$f_{\theta}\left( x \right)=\frac{(c-1)f_{\theta}^{*}\left( x \right)}{c-f_{\theta}^{*}\left( x \right)}$$

where

$$c=\frac{Pr\left( y=1|s=1 \right)}{Pr\left( z=1|s=1 \right)}.$$

**SUPPLEMENTARY REFERENCES**

[1] Ward, G., Hastie, T., Barry, S., Elith, J. and Leathwick, J. R. Presence‐only data and the EM algorithm. *Biometrics*, 65, 2 (2009), 554-563.

[2] Yang, P., Li, X.-L., Mei, J.-P., Kwoh, C.-K. and Ng, S.-K. Positive-unlabeled learning for disease gene identification. *Bioinformatics*, 28, 20 (2012), 2640-2647.

[3] Khan, S. S. and Madden, M. G. One-class classification: taxonomy of study and review of techniques. *The Knowledge Engineering Review*, 29, 3 (2014), 345-374.

[4] Yang, P., Liu, W. and Yang, J. *Positive unlabeled learning via wrapper-based adaptive sampling*. City, 2017.

[5] Wang, C., Ding, C., Meraz, R. F. and Holbrook, S. R. PSoL: a positive sample only learning algorithm for finding non-coding RNA genes. *Bioinformatics*, 22, 21 (2006), 2590-2596.

[6] Calvo, B., Larrañaga, P. and Lozano, J. A. Learning Bayesian classifiers from positive and unlabeled examples. *Pattern Recognition Letters*, 28, 16 (2007), 2375-2384.

[7] Joachims, T. *Transductive inference for text classification using support vector machines*. City, 1999.

[8] De Bie, T., Tranchevent, L.-C., Van Oeffelen, L. M. and Moreau, Y. Kernel-based data fusion for gene prioritization. *Bioinformatics*, 23, 13 (2007), i125-i132.

[9] Li, W., Guo, Q. and Elkan, C. A positive and unlabeled learning algorithm for one-class classification of remote-sensing data. *IEEE Transactions on Geoscience and Remote Sensing*, 49, 2 (2010), 717-725.

[10] Munroe, D. T. and Madden, M. G. Multi-class and single-class classification approaches to vehicle model recognition from images. *proc. AICS* (2005), 1-11.

[11] Manevitz, L. M. and Yousef, M. One-class SVMs for document classification. *Journal of machine Learning research*, 2, Dec (2001), 139-154.

[12] Yu, H., Han, J. and Chang, K. C.-C. *PEBL: positive example based learning for web page classification using SVM*. City, 2002.

[13] Liu, B., Lee, W. S., Yu, P. S. and Li, X. *Partially supervised classification of text documents*. Citeseer, City, 2002.

[14] Liu, B., Dai, Y., Li, X., Lee, W. S. and Yu, P. S. *Building text classifiers using positive and unlabeled examples*. IEEE, City, 2003.

[15] Li, X. and Liu, B. *Learning to classify texts using positive and unlabeled data*. City, 2003.

[16] Nigam, K., McCallum, A. K., Thrun, S. and Mitchell, T. Text classification from labeled and unlabeled documents using EM. *Machine learning*, 39, 2-3 (2000), 103-134.

[17] Lee, W. S. and Liu, B. *Learning with positive and unlabeled examples using weighted logistic regression*. City, 2003.

[18] Song, H. and Raskutti, G. PULasso: High-dimensional variable selection with presence-only data. *Journal of the American Statistical Association* (2019), 1-30.

[19] Breiman, L. Bagging predictors. *Machine learning*, 24, 2 (1996), 123-140.

[20] Mordelet, F. and Vert, J.-P. ProDiGe: Prioritization Of Disease Genes with multitask machine learning from positive and unlabeled examples. *BMC bioinformatics*, 12, 1 (2011), 389.

[21] Mordelet, F. and Vert, J.-P. A bagging SVM to learn from positive and unlabeled examples. *Pattern Recognition Letters*, 37 (2014), 201-209.

[22] Claesen, M., De Smet, F., Suykens, J. A. and De Moor, B. A robust ensemble approach to learn from positive and unlabeled data using SVM base models. *Neurocomputing*, 160 (2015), 73-84.

[23] Yang, P., Humphrey, S. J., James, D. E., Yang, Y. H. and Jothi, R. Positive-unlabeled ensemble learning for kinase substrate prediction from dynamic phosphoproteomics data. *Bioinformatics*, 32, 2 (2016), 252-259.

[24] Yang, P., Ormerod, J. T., Liu, W., Ma, C., Zomaya, A. Y. and Yang, J. Y. AdaSampling for positive-unlabeled and label noise learning with bioinformatics applications. *IEEE transactions on cybernetics*, 49, 5 (2018), 1932-1943.
